# Course Design for College Entrepreneurship Education – From Personal Trait Analysis to Operation in Practice

**DOI:** 10.3389/fpsyg.2019.01016

**Published:** 2019-06-04

**Authors:** Hsin-Te Wu, Mu-Yen Chen

**Affiliations:** ^1^Department of Computer Science Information Engineering, National Penghu University of Science and Technology, Magong, Taiwan; ^2^Department of Information Management, National Taichung University of Science and Technology, Taichung, Taiwan

**Keywords:** entrepreneurial education, entrepreneurial development, entrepreneurship and innovation management, innovation, personal trait analysis

## Abstract

Nowadays, many countries are promoting entrepreneurial education or the “innovation, entrepreneurship, and creativity” education. Entrepreneurial education can enhance a nation’s economic competitiveness and give rise to new business. At the moment, entrepreneurial courses are mostly designed by school teachers; however, while school teachers may possess business experience, they lack in entrepreneurial experience. Hence, entrepreneurial education courses call for experts with entrepreneurial experience to contribute to course designs and assist with course teachings. Entrepreneurial education not only improves a student’s entrepreneurial skills, but also enables each student to explore their personal characteristics in order to advance the collaboration efficacy of the team as a whole. This study asked six experts with entrepreneurial experience in the information industry to work with school teachers in course design as well as teaching collaboration. The course design starts with three talk sessions given by professionals who share with students their thoughts and experiences in entrepreneurial products, team organization, fund raising, and profit calculation. Following that, each student is asked to share their own thoughts on entrepreneurial products and start searching for team members and planning their project. During the course, each team receives six individual advising sessions from the professionals, with topics ranging from product modeling, feasibility, product market estimation, fundraising methods, and profit calculation. The experts also provide each team member with personal trait analysis. Last but not least, the course invites five management-level industry professionals to play the role of venture capital investor, and evaluate each team’s product modeling based on their presentation. This study reviews the grades given by the experts as well as the evaluations given by the three industry managers to assess whether the entrepreneurial education course’s student entrepreneur teams satisfy the industry’s expectations.

## Introduction

The modern technology industry is in rapid development and often demands sizable financial input toward product development; among them, many successful stories started in campuses with students forming teams and taking up entrepreneurial activities (Jones and Liu, 2017), notably Microsoft and Google. Information technology (IT) products require great amount of funding for the development and marketing processes; however, students can hardly meet such financial demand, which is why they often rely on venture capital from private firms or crowdfunding. Pebble Watch is among one of such examples. In the past, school education focused on fostering students’ professional skills or academic capabilities (Medina et al., 2018), and students had no access to entrepreneurial training. As the world grows more competitive, firms that in the past constituted mainly of field-specific professionals are nowadays in need of employees that not only have professional skills but also possess entrepreneurial talent (Troudt et al., 2017). Meanwhile, given the Information Age’s rapid growth, schools should help students develop teamwork and communication skills so as to satisfy the needs of their future career positions.

Entrepreneurial education was the main topic of concern in the European Commission’s, 2012 developmental policies (European Commission, 2012). Application of entrepreneurial concepts can help resolve issues of resource allocation in certain underprivileged areas as well as improve unemployment rates. Moreover, applying entrepreneurial concepts can help make aspects of our daily lives or the city we live in more innovative, such as art, automobile, and quality of life. After being trained through the entrepreneurial education program, workers will be able to come up with more innovative ideas at their jobs, and this will bring room for change for our society – which is also one of the “renaissance of entrepreneurship” goals set by the European Commission (Council of the European Union, 2014). Currently, many colleges across different nations have included entrepreneurial education in their curriculum. The design and teaching of such courses call for joint participation from experienced entrepreneurs. On one hand, the businessmen can fulfill their societal responsibilities (Elena and Timo, 2015; Lindner, 2018). On the other hand, while school teachers may have professional skills and industry experiences, they lack in entrepreneurial experience; hence, they need the joint effort of businessmen with entrepreneurial experience to help them with teaching. Incorporating entrepreneurial education into courses can motivate the students’ entrepreneurial spirits and cultivate a spirit of societal responsibility (Mehta et al., 2018). In an startup team, each member has a role to play. The entrepreneurial course not only teaches students entrepreneurial capabilities but also explores their potentials and personal traits and then trains them accordingly (Neumeyer and McKenna, 2018). Students can learn about the roles they play in the team as well as their own potentials and strong suits, and the course’s training enhances their ability to deal with challenges in their future careers.

This study aims at investigating how the proposed entrepreneurial course is received within the industry and whether it is helpful toward students in helping them explore their personal traits. Additionally, the study conducts a course design satisfaction analysis using questionnaires. The proposed course was designed by school teachers and experts with entrepreneurial experiences. Experts share their entrepreneurial journey during three talk sessions, during which students can start to entertain their own startup ideas and analyze traits they want to look for in team members. Following that, the school teachers will ask the students to prepare presentations and assemble a team. The experienced experts are invited to serve as co-advisors for the students by taking turns in teaching each team all the steps along the way. Finally, the experts are asked to grade each team’s project. The effect of the proposed course design is verified by taking a look at the students’ project results: the study compares the grades given by the experienced experts during each advising session with the overall grades at the end of the term to assess whether each team’s startup success rate is believable. The tools used for verification is statistical product and service solutions (SPSS). SPSS is used to analyze the composite reliability aspect while course evaluation forms can help assess the students’ reception toward the overall course arrangement. Analysis results suggest that by working with experts in the industry, students receive better training in the entrepreneurial course and gain recognition from business enterprises.

### Problems of Research

After unemployment rates skyrocketed following the global financial crisis, many youngsters began dreaming of starting their own business. According to Hsiao (2016), the success rate of youngsters starting their own business is 10%, and the biggest obstacle in their entrepreneurial attempt lies in financial funding – something that can crush their dreams altogether. Even with the most ambitious entrepreneurial intentions, the youth may not success, and the biggest reason is their lack of training in entrepreneurship-related courses (Li and Yang, 2011). Therefore, many universities have started offering entrepreneurial courses to educate their students in entrepreneurial knowledge. It is most fitting for universities to incorporate entrepreneurial education into their curriculum because since the goal of universities is to cultivate professional skills and academic capabilities, adding entrepreneurial professionalism would go a long way in helping students create new career opportunities. When incorporating entrepreneurial education into the curriculum, universities must take note of their teaching approach and course design (Strachan, 2018). The practices of entrepreneurial education must fall in line with the Education for Sustainable Development (UNESCO, 2016) in order to adhere to the educational goal set by the United Nations. College courses should move from the classroom teaching approach of the past toward diversified approaches. In the past, college education focused on fostering professional skills; nowadays, it must incorporate entrepreneurial education. Nevertheless, problems may arise in four aspects: (1) unreasonable course design; (2) lack of entrepreneurial experience in teachers; (3) entrepreneurial education teaching approach; and (4) inadequate structural planning in the entrepreneurial education system (Weiming et al., 2016).

Aside from teaching students how to start a business, entrepreneurial education also explores the students’ intentions for entrepreneurship. In Hamidi et al. (2008), industry experts share entrepreneurial experiences with students, and their experiment results suggest that entrepreneurial training can enhance entrepreneurial intention in students. Johansen (2014) examined the relationship between the student’s academic performance and entrepreneurship and concluded that the two factors were irrelevant to each other. Meanwhile, Neumeyer and McKenna (2018) utilized entrepreneurial education to teach students about the importance of division of labor in teamwork so as to elevate their personal skills and abilities in the role the play in the team. Edokpolor and Somorin (2017) illustrated that entrepreneurial education needs to cultivate talents that demonstrate competence in entrepreneurship, productivity, ability to innovate, analytical skills, proactivity, and interpersonal relationship abilities. Given the above, entrepreneurial education requires a series of courses to develop the student’s personal traits.

Seikkula-Leino et al. (2015) mentions 30 partners in a school that worked together toward providing entrepreneurial environment and courses for their entrepreneurial education, and their teaching strategies were widely praised. In Lahn (2016), students were sent to work as interns at business firms and learn about entrepreneurship through the experience while in Nielsen and Stovang (2015), entrepreneurial education took the form of “about,” “for,” “through,” and “embedded” so as to enable students to gain entrepreneurial skills. On the other hand Karimi et al. (2016), discusses entrepreneurship and entrepreneurial opportunities. It talks about how to train students not only in entrepreneurship but also in creating entrepreneurial opportunities for themselves. Moreover, it instructs students on how to go from brainstorming to conceptualizing their startup ideas, increasing their chances of realizing such ideas. By contrast, Baschiera et al. (2018) discuss how today’s educators lack entrepreneurial capabilities and thus need input and collaboration from industry businessmen to help better the students’ entrepreneurial skills. On this note, Belitski (2016) argues that students’ entrepreneurial skills can only be nurtured through the collaborative work between the university, the industry, and the government. Duval-Couetil et al. (2014) collected demographic characteristics to analyze the students’ entrepreneurial intentions. Riese (2013) sent students to small-sized companies to intern and start their own business, gaining actual, hands-on experience in entrepreneurship. Rossano et al. (2016) incorporated problem-based learning (PBL) with entrepreneurial education – teachers who used to play the part of simply passing on knowledge must now take on the role of guiding students in team learning. Duval-Couetil et al. (2016) notes that, in an era of swift technological development, engineering majors may find entrepreneurial opportunities more easily if they also partake in entrepreneurial courses. The entrepreneurial courses are conducted in an experiential manner and elevates the students’ entrepreneurial skills.

### Research Focus

This study incorporated entrepreneurial education into the course; however, it ran into the following problems: (1) While many college professors have experience working in the industry, they seldom have entrepreneurial experience. (2) College professors are dedicated toward teaching academic or professional subjects, and they lack in teaching entrepreneurial courses. (3) College professors do not have access to entrepreneurial resources and, as a result, cannot provide students with the funds or funding approaches in their entrepreneurship. (4) Entrepreneurial courses should not only teach entrepreneurial skills but also allow students to learn about their personal traits in teamwork. The proposed course design has the following purposes: (1) Having industry experts with entrepreneurial experience join in planning the coursework makes the course more comprehensive. (2) Allowing expert instructors to focus on entrepreneurial teaching while college professors focus on academic and professional subjects allows students to enjoy all-around learning about entrepreneurship and realize product development. (3) Introducing industry experts to the classroom gives the students a chance to learn all about fund raising; meanwhile, the expert instructors are invited to grade the startup teams’ projects at the end of the term, increasing chances of collaboration between the students and the businesses. (4) Instructors will offer reviews to each team during each stage of the course, allowing individual students to understand their own traits better.

## Materials and Methods

The proposed method invites experts in the professional field to cohost the courses. This not only offers students a chance to improve their hands-on entrepreneurial skills but also allows them to make further contact with the business industry and perhaps gain access to business resources. Likewise, college professors can benefit by gaining entrepreneurial experience. This section provides an elaboration of the overall course design, collaboration approach with industry experts, the student teams’ grading method, and the business firms’ grading method.

### The Overall Course Design

**FIGURE 1:**
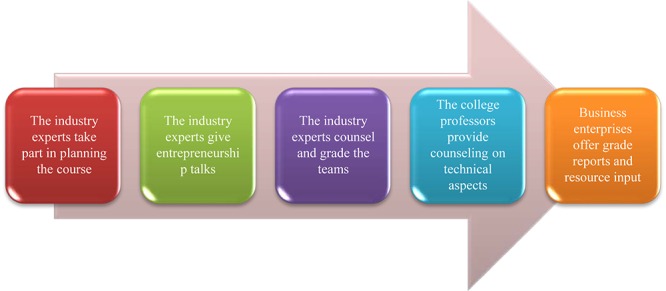
Execution procedure of the entrepreneurial course.

The proposed entrepreneurial course’s course planning procedure is shown in Figure 1. The course invites industry experts with entrepreneurial experience to join in planning the course. Most students who take this course are computer science majors; the industry experts mostly have some background in computer science. There will be three entrepreneurship talk sessions that hope to help students understand the entrepreneurial process and cultivate the necessary skills needed during the process of starting a business. After the talk session, students will begin to look for their teammates and start conceptualizing their product as well as prepare PowerPoint presentations. Next, the industry experts will offer adjustment advice to each team based on their division of labor and product presentation. The expert instructors will regularly provide guidance and grade reviews; meanwhile, the role of the college professors is to advise on the technical aspect. Moreover, during the time of the industry experts’ participation, college professors can listen, and learn about entrepreneurial experiences to facilitate their future promotion of related courses. Each time the industry expert advises on the teams, they will give concrete advice and scores to the team’s labor of division, which the college professors may then utilize to have the teams make adjustment. Additionally, the received advice can make college professors aware of each student’s strengths and weaknesses so as to offer further guidance to fortify each student’s specialty in the team. After the students devise the product modeling, the experts will give each team a grade based on the model and offer subsequent funding or resource information. The industry expert grading mechanism gives the business enterprises a chance to know each startup team and even match them up.

### Industry Expert Collaboration Approach

For the course design of the proposed entrepreneurial course, college professors invite industry experts with entrepreneurial experience to jointly plan and design the coursework. In this study, six industry experts took part in the course design following this procedure:

(1) Three entrepreneurship talk sessions were offered. The first talk session focused on entrepreneurial concepts, the second focused on entrepreneurial skills, and the third one discussed startup funding.
(2) The industry experts conducted six individual advising sessions for each team. Each expert advised teams separately so that the students could learn about actual entrepreneurial process from their team’s presentation and product modeling. On the other hand, the experts could correct the students’ mistakes during the process and improve their entrepreneurial process.
(3) The college professors learned about entrepreneurial skills and concepts during the course, which compensated their ability to teach entrepreneurial courses. In the future, when they offer related courses, they will be able to design, and teach a course by themselves.

Each talk session lasted for 3 h. The first session, a talk on entrepreneurial concepts, focused on discussing entrepreneurial risks and entrepreneurial concepts to teach students that entrepreneurship requires more than technical skills – it involves innovation, creativity, and feasibility. While the first talk offered students entrepreneurial concepts, the second talk advised them on product positioning. It taught them how to analyze the product’s target market, the product’s advantages, demands, and profit calculation. The third talk involved startup funding, which mostly concerned presentation, funding, and venture capital concepts, teaching students how to obtain startup funds through fundraising or venture capital investments.

Each industry expert took turn in counseling the student teams, advising them on aspects such as the product itself, market positioning, and product advantage. They also offered comments on each team members role in the team to teach students about hands-on entrepreneurial skills and abilities. The college professors, who lack in entrepreneurial experience, could utilize their participation in the course to absorb entrepreneurial experience, and which would become helpful toward when they offer related courses in the future. Ideally, the college professors will be able to work alone in setting up course design and teaching the course.

### Student Grading Mechanism

During the proposed entrepreneurial course, the industry experts take turns in giving each student team counseling sessions and grading reports, as shown in Table 1. The experts will give grades during each of the advising sessions; the grading criteria was decided by the six industry experts after some discussion. The first parameter is target market planning, which assesses the startup team’s product market share. This parameter helps investors understand the product’s future developments. The second parameter is product feasibility, which concerns the product’s realization. It prevents the product from being too unrealistic or impractical and assesses whether the conceptualization is reasonable. The third parameter is profit calculation. The team is asked to analyze the expected return on investment (ROI) of the product after its launch into the market. The fourth parameter is team performance. This is an evaluation of each team member’s role in their teamwork, including each member’s execution progress. Last but not least, the fifth parameter concerns financial planning. It evaluates the funds needed for the team’s product as well as how reasonable the team is with its use of funds during business operation. Finally, in the “comment” section, the industry experts offer variegated opinions. They will also point out any mistakes during the team’s presentation to allow students to improve their entrepreneurial and personal abilities from hands-on operations. The college professors can take the report from each expert counseling session and ask students to make adjustments accordingly. Each report is also passed on to the next expert advisor; in this way, the industry expert can not only have a grasp of each team’s issues from the previous time, but they can also follow up on the progress. The proposed entrepreneurial course also established industry expert chat groups on social media websites to help them closely monitor and discuss the student teams’ progress.

**Table 1:** Grade report form for industry experts.

Parameter\team	Target market planning (20%)	Product feasibility (25%)	Product profit calculation (20%)	Team performance (20%)	Financial planning (15%)	Total score
Comments						

### Business Firm Grading Mechanism

This course invited three management-level industry professionals to grade the team’s venture capital projects with the grading report shown in Table 2. The business firm grading mechanism focuses on reviews from business investors. For instance, for the first parameter of target market planning and competitiveness, the firm will take into consideration the product’s feasibility, and competitiveness in a real market. The second parameter concerns product feasibility and marketing strategies. This takes into consideration the product’s viability and its future marketing strategies for the market. Even if a product is feasible, the marketing of the product may be even more labor- and fund-consuming, which is why the startup team must address their marketing strategies. The third parameter is business model and financial planning, which mainly serves to help investors understand the startup team’s future business model as well as their financial planning. The team must also address their product’s expected ROI, and the business professionals will evaluate whether the startup team’s financial plans are reasonable and how they fare in the product’s ROI. The fourth parameter is team performance, in which the business professionals evaluate each team’s professionalism, and their performance across different aspects. Team performance is also the highlight of the whole startup project. Lastly, the business professionals offer advice after listening to each team’s presentation and Q&A session. They also include their comments in the grade reports.

**Table 2:** Grade report form for management-level industry professionals.

Parameter\team	Target market planning and competitiveness (25%)	Product feasibility and marketing strategies (25%)	Business model and financial planning (25%)	Team performance (25%)	Total score
Comments						

## Results

This section includes two subsections: grade report results and discussion.

### Grading Result Analysis

This study utilized the statistical analysis software – statistical package for social science (SPSS) – to run analysis of variance on the grades given to the students by the industry experts and business professionals. The course given in this study was titled “Internet of Things Design Course;” a total of 38 students enrolled in the course and were subsequently divided into 12 teams to undergo entrepreneurial training. The study employed the distribution-free statistical method in determining the reliability of grades. The *z*-score was used to assess whether the industry experts and business professionals agree on the grades to each team. Table 3 is a *z*-score analysis of the six industry experts’ grades toward the twelve student teams while Table 4 is a calculation of whether there is significant variance between grades offered by different industry experts. Both the *z*-score of Tables 3, 4 fall in the confidence interval between -2 and +2, indicating that the industry experts gave consistent grades to each team. Meanwhile, Table 5 is a *z*-score analysis of the three business professionals’ grades toward the twelve student teams while Table 6 is a calculation of whether there is significant variance between grades offered by different business professionals. It is seen from Tables 5, 6 that both of their *z*-score fall in the confidence interval between -2 and +2, indicating that the business professionals offered consistent grades to each team. It can be concluded from Tables 3–6 that all the industry experts and business professionals offered consistent grades to the students’ startup products. If multiple industry experts and instructors offer positive feedback, then this means that the product proposed by the entrepreneurial team is feasible and applicable in the real-world industry. This study’s proposed method allows entrepreneurial education to be realized in a college curriculum; the course approach can effectively integrate professional skills with entrepreneurship. Entrepreneurial education can enhance students’ motivation toward learning the courses; as shown in Table 7, students showed a satisfaction rate of 4.39. By having business experts and college professors to jointly offer the course helps students as well as the professors to improve their professional abilities and entrepreneurial experiences; moreover, it achieves the goal of encouraging/realizing industry-academy cooperation as well as bridging the gap between theory and practice.

**Table 3:** *Z*-score statistics of the grades given by the industry experts.

Student team	Mean	Standard deviation	*Z*-Score
1	63.28	7.56	-1.89
2	76.38	5.62	0.16
3	70.73	4.68	-0.78
4	83.83	5.24	-0.71
5	86.23	4.98	-0.35
6	53.54	11.29	-0.49
7	87.86	3.10	-0.28
8	59.75	8.70	-1.37
9	84.38	5.00	-0.87
10	62.69	5.20	-1.08
11	64.33	7.88	-0.52
12	52.42	17.79	-0.67

**Table 4:** *Z*-score statistics of the grades given by each industry expert.

Industry expert	Mean	*Z*-Score
1	64.89	-1.03
2	70.96	0.09
3	63.15	-1.36
4	72.46	0.37
5	76.83	1.18
6	74.43	0.74

**Table 5:** *Z*-score statistics of the grades given by the business professionals.

Student team	Mean	Standard deviation	*Z*-score
1	65.50	4.38	-0.97
2	71.92	2.70	-0.43
3	73.67	4.37	-0.27
4	86.00	3.88	-0.97
5	88.08	2.01	-1.04
6	67.42	6.52	-1.14
7	88.33	2.89	0.58
8	61.08	10.17	-1.09
9	87.92	1.84	-0.77
10	67.58	6.71	-1.13
11	70.58	1.46	-0.74
12	64.92	5.00	-0.98

**Table 6:** *Z*-score statistics of the grades given by each business professional.

Business professional	Mean	*Z*-score
1	70.73	-1.15
2	76.67	0.70
3	75.85	0.45

**Table 7:** Teaching evaluation of the entrepreneurial education course.

Question number	Question	Average
**The instructor’s teaching techniques (0–5, 5 being the highest score)**		4.43
01	The instructor was able to provide a comprehensive syllabus (including teaching goals, progress, and grading criteria)	4.41
02	The instructor’s teaching contents were organized and focused	4.44
03	The instructor demonstrated professional knowledge in the subject taught	4.44
04	The instructor was well prepared	4.46
05	The instructor was enthusiastic and dedicated toward teaching	4.44
06	The instructor was never late or absent to class or modified class schedule with no good reason	4.41
07	The instructor was able to motivate the student’s learning interests	4.44
08	The instructor’s lectures were clear and cohesive in a way that was easy to understand	4.41
09	The instructor gave tests that evaluated the students in an effective manner	4.39
10	The instructor graded students in an objective and fair manner	4.41
**The student’s self-evaluation (0–5, 5 being the highest score)**		4.37
11	I believe that the course was offered with great teaching quality	4.39
12	I am satisfied at the instructor’s teaching during the course	4.34
13	I feel like I learned a lot from taking the course	4.37
14	I would recommend this course to my junior classmates	4.37
Overall average score		4.4

## Discussion

The proposed entrepreneurial course employed the approach of inviting industry experts to join in course design and teaching. This helps students understand current entrepreneurial skills and industry resources. The proposed scheme aims at enriching the professors’ experiences in entrepreneurial courses while also cultivating the students’ entrepreneurial abilities. Entrepreneurial education has become an educational goal in many different countries. The purpose of inviting industry experts to join in course design and teaching is to elevate the course’s practicality; meanwhile, having multiple industry experts jointly offer a course broadens the students’ scope of learning experience. Having different instructors assess and grade the products of the entrepreneurial teams also raises the reliability of such gradings. Finally, the study invited several management-level business professionals to give the entrepreneurial teams an overall grading, the goal of which was to utilize the perspective of these management-level professionals to assess the success rate of such entrepreneurial products. Last but not least, the experiment results served to verify whether the course design did indeed meet the demands of management-level professionals in the industry. The purpose of bringing industry professionals into the project is to create collaboration between business experts and entrepreneurial teams so that the entrepreneurial teams in the course may genuinely create a product and truly realize the process of entrepreneurship.

## Conclusion

The proposed entrepreneurial course was jointly designed by college professors and industry experts. The course devised three entrepreneurship talk session to teach share with students experiences in entrepreneurial skills and resources. The industry experts were in charge of teaching entrepreneurial skills while the college professors were in charge of cultivating technically professional skills; finally, the business professionals took upon the task of grading each team’s product, allowing students to learn about the entrepreneurial process as well as their product’s strengths, and weaknesses while also elevating their entrepreneurial capabilities and professional competitiveness. The proposed entrepreneurial education course can achieve the following goals: (1) enrich college professors’ experiences with entrepreneurial education; (2) incorporate industry resources and bridge the gap between theory and practice; (3) jointly design the course with the industry to ensure cultivation of talents needed in the real-world industry; (4) raise the entrepreneurial teams’ success rate by having both the instructors and management-level business professionals offer their assessment; and (5) encourage and facilitate industry-academy cooperation opportunities by inviting industry professionals to jointly offer the course. The proposed procedure offers a break-through from past traditional teaching approaches by having both business experts and management-level professionals teach and grade the students, all the while offering entrepreneurial experiences and resources that can help improve the chances of success for the entrepreneurial teams.

## Ethics Statement

An ethics approval was not required as per applicable institutional and national guidelines and regulations. The informed consent of the participants was implied through participation.

## Author Contributions

H-TW presented the research topic and concept, and designed and implemented the experiments. M-YC surveyed the literatures and research methodologies, finished the experimental process, and discussed the experimental results.

## Conflict of Interest Statement

The authors declare that the research was conducted in the absence of any commercial or financial relationships that could be construed as a potential conflict of interest.
